# Exploring the Therapeutic Potential of Apigenin in Obesity-Associated Fibrinolytic Dysfunction: Insights From an Animal Study

**DOI:** 10.7759/cureus.40943

**Published:** 2023-06-25

**Authors:** Hasan H Qadi, Mohamed A Bendary, Safa Y Almaghrabi, Mohammed Alameen F Zaher, Mohamed M Karami, Ahmed M Alsehli, Omar Babateen, Ahmad F Arbaeen, Abdulhadi S Burzangi, Mohammed A Bazuhair

**Affiliations:** 1 Department of Clinical Physiology, Faculty of Medicine, King Abdulaziz University, Jeddah, SAU; 2 Department of Physiology, Faculty of Medicine, Umm Al-Qura University, Makkah, SAU; 3 Department of Laboratory Medicine, Faculty of Applied Medical Sciences, Umm Al-Qura University, Makkah, SAU; 4 Department of Clinical Pharmacology, Faculty of Medicine, King Abdulaziz University, Jeddah, SAU

**Keywords:** apigenin, plasminogen activator inhibitor-1, fibrinolysis, inflammation, oxidative stress, obesity

## Abstract

Introduction: Obesity (Obe) is a chronic metabolic disorder usually complicated by impaired fibrinolytic activity. Apigenin (Api) is one of the flavonoids that have anti-adiposity effects. This study aimed to explore the therapeutic potential of Api in high-fat diet (HFD)-induced obese rats.

Methods: Twenty-four Wistar adult male rats were randomly allocated into control group, supplemented with a normal pellet diet (NPD); Api group, supplemented with Api (10 mg/kg) for eight weeks; Obe group, obesity was induced by feeding HFD for eight weeks; and Obe/Api group, obese rats supplemented with Api for eight weeks. Body mass index (BMI), homeostatic model assessment of insulin resistance (HOMA-IR), tumor necrosis factor-α (TNF-α), malondialdehyde (MDA), total superoxide dismutase (t-SOD) activity, and plasminogen activator inhibitor-1 (PAI-1) were measured.

Results: Compared to the control group, Obe group exhibited a significant increase in BMI, HOMA-IR, TNF-α, MDA, and PAI-1. These results were also associated with a significant decrease in serum t-SOD activity. Supplementation of Api alleviated the measured deteriorated parameters and ameliorated visceral adiposity in obese rats.

Conclusion: This study provides compelling evidence regarding a promising role for Api in ameliorating the impairment of fibrinolytic activity in an Obe animal model. The observed effects are likely mediated through Api's anti-obesity properties, as well as its indirect modulation of PAI-1, oxidative stress, and inflammation. Future clinical studies are recommended that may make benefit of the preclinical therapeutic use of apigenin in obesity-associated fibrinolytic dysfunctions.

## Introduction

The global prevalence of obesity (Obe) has increased dramatically over the past few decades and has become a pandemic in not only developed but also developing countries [1]. Furthermore, Obe is usually associated with impaired fibrinolytic activity that is attributed mainly to increased plasminogen activator inhibitor-1 (PAI-1), which is the major inhibitor of the fibrinolytic system [2]. Also, PAI-1 inhibits tissue-type plasminogen activator (t-PA) and urokinase-type plasminogen activator (u-PA) [3,4].

There is much-emerging evidence that Obe is associated with increased blood PAI-1 levels which is a risk factor for higher incidence of coronary heart disease and cardiovascular thrombotic events. Also, with obesity, the higher level of PAI-1 hinders the breakdown of clots and indirectly contributes to their formation [5-7]. The pathogenesis of fibrinolytic dysfunction in obesity is correlated to body mass index (BMI), insulin resistance (IR), oxidative stress, and low-grade inflammation [8,9].

Recently, attention was paid to the prevention of obesity-associated fibrinolytic dysfunction by using natural compounds, such as “flavonoids” [10,11]. Apigenin (Api) is one of the flavonoids that is present in vegetables and fruits, e.g., parsley, celery, onions, and oranges [12]. It has been shown that Api exhibits anti-inflammatory, antioxidant, and anti-adipogenic effects [13-15]. Therefore, by curbing the accumulation of adipose tissue, Api could contribute to a decrease of PAI‐1 and hence indirectly lessen the likelihood of clot formation. Moreover, Api inhibits platelet adhesion and thrombus formation [16]. Therefore, this work was designed to elaborate on the potential role of this flavonoid in ameliorating Obe-associated fibrinolytic dysfunction in an animal model.

## Materials and methods

Diet and treatment

Apigenin, a light yellow powder with purity >98% (CAS no: 520-46-5) was purchased from Sigma-Aldrich Co. (St. Louis, MO). High-fat diet (HFD) was freshly prepared every three days. It consisted of 43% carbohydrates, 17% protein, and 40% fat and provides 414 kcal/100 g. HFD was reconstituted in a mixture of 68% normal pellet diet (NPD), 6% corn oil, 6% ghee, and 20% instant milk powder [17].

Experimental protocol

Twenty-four adult male Wistar rats weighing 175±25 g and aged three months were left to acclimatize for one week before the start of the experiments. They were housed at an ambient temperature of 23±2°C, under the natural 12-hour day/night cycle with free access to food and tap water. The experimental protocol of this study was approved by the local Ethical Committee at the Faculty of Pharmacy, King Abdulaziz University, Jeddah, Saudi Arabia. The local institutional rules were in strict accordance with the international guiding principles for the care and use of laboratory animals.

The rats were divided into four following groups: control group, rats in this group were supplemented with NPD for eight weeks. Api group, rats were administered orally with Api (10 mg/kg) dissolved in 0.1% dimethyl sulfoxide (DMSO) for eight weeks. Obe group, rats supplemented with HFD for eight weeks [18]. Obe/Api group, obese rats were administered with Api (10 mg/kg) for eight weeks. Body mass index was calculated following this formula; BMI = body weight (g)/length (cm^2^) [19].

Serum sample collection

Following the diet and treatment period morning fasting blood samples were withdrawn from the retro-orbital venous sinuses and the blood was centrifuged at 7,000×g for five minutes in a microcentrifuge (1-15P microfuge, Sigma Microfuge, Shropshire, UK: SciQuip Ltd) and serum was separated and stored at -20°C until biochemical analysis.

Biochemical analysis

Serum glucose measurement was performed using a commercial glucose assay kit following the manufacturer's instructions. Serum insulin and TNF-α measurement were determined using rat-specific ELISA kits (San Diego, CA: MyBioSource), according to the provided protocol. Serum PAI-1 measurement was determined using a rat-specific ELISA kit, following the manufacturer's instructions. Serum MDA and t-SOD measurement - MDA, a marker of oxidative stress, and t-SOD, an indicator of the antioxidant defense system, were measured using colorimetric assay kits, following the respective protocols provided by the manufacturer. All measurements were performed in triplicate to ensure accuracy and reliability of results.

Measurement of insulin resistance

The gathered data of glucose and insulin were used to evaluate insulin resistance using the homeostatic model assessment of insulin resistance (HOMA-IR) that was calculated using the following formula: HOMA-IR = (fasting glucose in mg/dL×fasting insulin in μIU/mL)/405 [20].

Statistical study

The results were analyzed using the SPSS software (Chicago, IL: SPSS 23 Inc.). The numerical variables were expressed as mean±SD and were checked for normality using the Shapiro-Wilk test. The statistical differences between groups were determined by one-way analysis of variance (ANOVA) followed by post-hoc test of least significant difference (LSD). P-value≤0.05 was considered statistically significant.

## Results

Effect of Api on BMI

**Figure 1:**
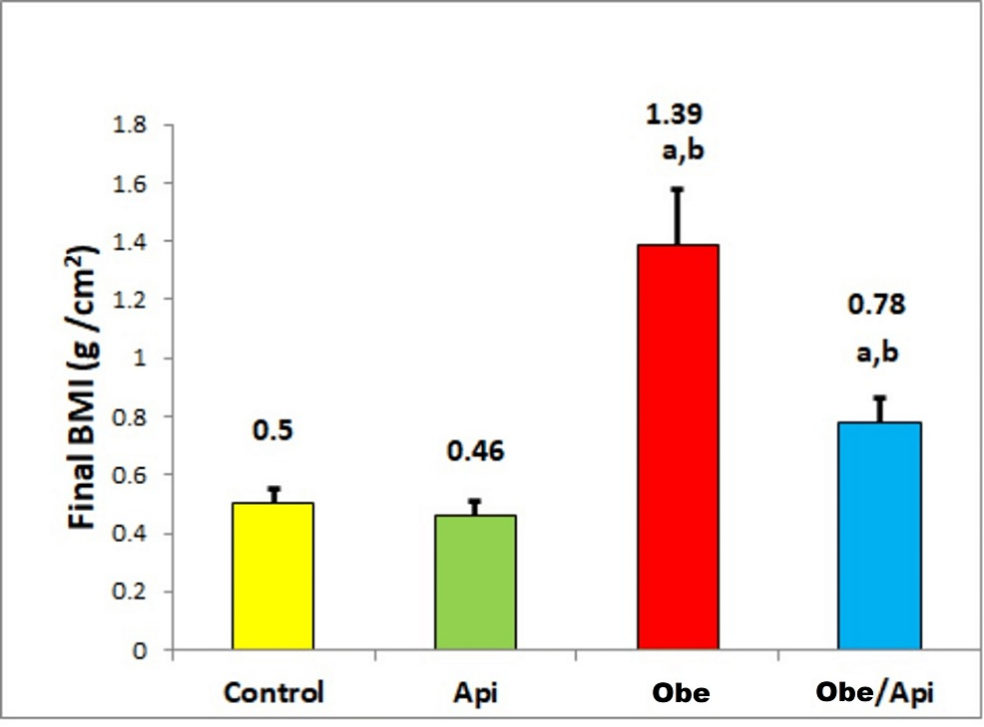
Final BMI in the control and experimental groups. ^a^P-value<0.05 when compared to control and Api groups. ^b^P-value<0.05 when compared to Obe group. Api: apigenin; Obe: obesity; BMI: body mass index The number of animals was six per group. Data are expressed as mean±SD.

After ingestion of HFD in the Obe group, there was a significant increase in the final BMI compared to the control and Api groups. In Obe/Api group, the final BMI was significantly lower compared to Obe group (Figure 1). 

Effect of Api on IR, inflammation, and oxidative stress

Ingestion of HFD resulted in significant increases in HOMA-IR, serum TNF-α, and MDA in the Obe group as compared to the control and Api groups. Also, Obe group demonstrated significant decrease in serum t-SOD activity. The addition of Api in the diet resulted in significant decrease in IR, serum TNF-α, and MDA. Furthermore, there was significant increase in serum t-SOD level in Obe/Api group as compared to Obe group (Table 1).

**Table 1:** HOMA-IR and serum TNF-α, MDA, and t-SOD in the control and experimental groups. ^a^P-value<0.05 when compared to control and Api groups. ^b^P-value<0.05 when compared to Obe group. Api: apigenin; Obe: obesity; HOMA-IR: homeostatic model assessment of insulin resistance; TNF-α: tumor necrosis factor-alpha; MDA: malondialdehyde; t-SOD: total superoxide dismutase, LSD: least significance difference Values are expressed as mean±SD. Statistical analysis was carried out using one-way ANOVA followed by LSD. The number of animals was six per group.

	Control	Api	Obe	Obe/Api	F-value	p-Value
HOMA-IR	4.95±0.53	4.13±0.42	13.60±1.39^a^	7.36±1.42^a,b^	1.93	0.015
TNF-a (pg/mL)	25.45±2.21	23.83±1.85	31.31±2.45^ a^	26.56±4.68^a^	6.83	0.002
MDA (nmol/mL)	6.25±1.08	6.08±0.86	21.96±3.87^ a^	13.78±1.82^a,b^	67.39	0.001
t-SOD (U/L)	165.00±10.41	163.66±5.92	133.83±8.61^a^	142.16±6.55^a^	22.381	0.02

Effect of Api on PAI-1

**Figure 2:**
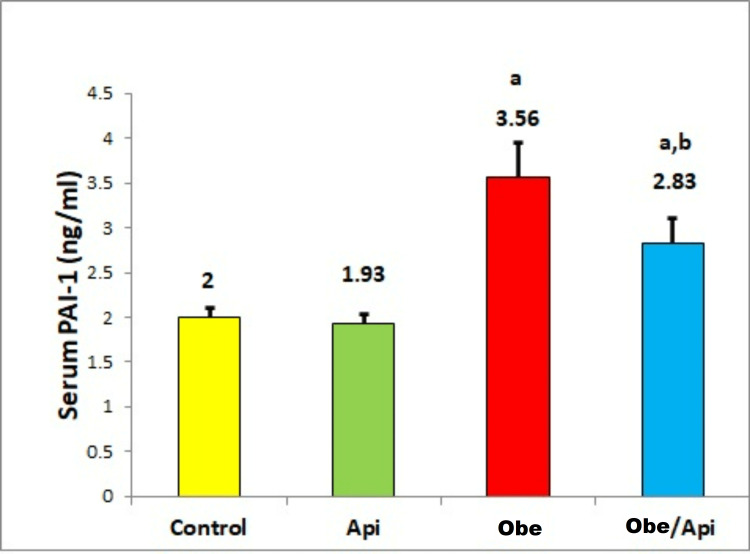
Serum PAI-1 in the control and experimental groups. ^a^P-value<0.05 when compared to control and Api groups. ^b^P-value<0.05 when compared to Obe group. Api: apigenin; Obe: obesity; PAI-1: plasminogen activator inhibitor one The number of animals was six per group. Data are expressed as mean±SD.

Obe group showed a significant increase in mean serum PAI-1 as compared to the control and Api groups. Supplementation with Api significantly decreased the mean PAI-1 in Obe/Api group when compared with Obe group (Figure 2).

## Discussion

Obesity is a serious metabolic disorder that is usually complicated in the long term with hemostatic complications, particularly fibrinolytic disorders. This work was settled down to elaborate on the potential therapeutic role of the flavonoid apigenin in ameliorating the fibrinolytic dysfunction in HFD-obese animal model.

In this study, Obe group exhibited a significant increase in BMI. This was concomitantly associated with a significant increase in IR. The later condition usually occurs when peripheral tissues like muscles, fat, and liver don't respond well to insulin hormone and hyperinsulinemia is a common squeal. Consequently, more lipogenesis is encountered by insulin that ultimately ends with elevations in BMI with a switch of cellular adiposity [21]. Adipocyte hypertrophy and hyperplasia are pathological features of obesity [22,23].

Obesity-induced IR is correlated positively with most of the obesity-associated complications, particularly, prothrombotic and hypofibrinolytic ones [24]. In that concern, in our results increased PAI-1 was associated with increased IR. A finding implied that the hypofibrinolytic milieu in obesity is related to IR.

The increase in BMI observed in the Obe group is commonly used as a diagnostic tool for characterizing generalized obesity. However, waist circumference (WC) represents a crude estimate of visceral obesity, which is associated with a higher risk of cardiometabolic complications [25,26]. In our study, we observed a significant decline in BMI following Api supplementation, suggesting an anti-obesity effect of this flavenoid. Moreover, Su et al. supported this finding by demonstrating the anti-visceral adiposity effect of Api through its impact on adipogenesis [15].

In this work, there was a significant increase in IR in the Obe group compared to the control groups. In obesity, various adipocyte-derived pro-inflammatory cytokines, including TNF-α, interleukin-1β, interleukin-6, monocyte chemotactic protein-1, leptin, and resistin are overproduced, contributing to IR [27,28]. Consistent with this, our study found a significant increase in serum TNF-α levels in the Obe group. This implied that obesity is associated with a state of systemic inflammation [29,30].

Also, Obe group displayed an increase in MDA and a decrease in t-SOD. Therefore, being a strong antioxidant flavonoid, Api ameliorated Obe-associated redox imbalance in Obe/Api group. Scientists have perpetuated that inflammation and oxidative stress are risk factors for Obe-associated disturbed fibrinolytic activity [31]. In this study, obese rats have encountered a significant elevation of serum PAI-1. This could be attributed to the concomitant rise of serum TNF-α in these rats. In alignment with this assumption, it was cited that PAI-1 synthesis is upregulated by TNF-α [32].

A previous study established a prothrombotic link between obesity and impaired fibrinolytic system in obese mice [4]. The high adipocyte-derived PAI-1 was documented as a risk factor for reduced fibrinolytic capacity, cardiovascular thrombotic events, higher incidence of coronary heart disease, and myocardial infarction [5-7]. In that regard, via a significant decrease in serum PAI-1 in Obe group, Api supplementation could indirectly prevent the previously mentioned obesity-associated thrombotic complications. Additionally, it was reported that Api, by unsettled mechanism, has the ability to inhibit platelet adhesion and thrombus formation [16].

## Conclusions

The findings of this study are unique in showing the ability of Api to alleviate fibrinolytic dysfunction associated with HFD-induced obesity. The confirmed mechanisms included decreased IR, inflammation, and oxidative stress associated with obesity. Enhanced fibrinolytic activity was depicted by the decrease in serum levels of PAI-1. Also, these findings confirmed that Api has anti-adipogenic, anti-inflammatory, and antioxidant properties. Further research is warranted to explore more underlying mechanisms and fully harness the therapeutic potential of Api in managing obesity-associated complications.
